# The microglial activation state regulates migration and roles of matrix-dissolving enzymes for invasion

**DOI:** 10.1186/1742-2094-10-75

**Published:** 2013-06-21

**Authors:** Starlee Lively, Lyanne C Schlichter

**Affiliations:** 1 Toronto Western Research Institute, Room MC9-417, 399 Bathurst Street, Toronto, ON M5T 2S8, Canada; 2 Department of Physiology, Medical Sciences Building, University of Toronto, 1 King’s College Circle, Toronto, ON M5S 1A8, Canada

**Keywords:** Alternative activation, Classical activation, LPS, IL-4, Cell migration, Cell invasion, Extracellular matrix degradation, Matrix-degrading enzymes, M1 polarization, M2 polarization

## Abstract

**Background:**

Microglial cells are highly mobile under many circumstances and, after central nervous system (CNS) damage, they must contend with the dense extracellular matrix (ECM) in order to reach their target sites. In response to damage or disease, microglia undergo complex activation processes that can be modulated by environmental cues and culminate in either detrimental or beneficial outcomes. Thus, there is considerable interest in comparing their pro-inflammatory (‘classical’ activation) and resolving ‘alternative’ activation states. Almost nothing is known about how these activation states affect the ability of microglia to migrate and degrade ECM, or the enzymes used for substrate degradation. This is the subject of the present study.

**Methods:**

Primary cultured rat microglial cells were exposed to lipopolysaccharide (LPS) to evoke classical activation or IL4 to evoke alternative activation. High-resolution microscopy was used to monitor changes in cell morphology and aspects of the cytoskeleton. We quantified migration in a scratch-wound assay and through open filter holes, and invasion through Matrigel™. A panel of inhibitors was used to analyze contributions of different matrix-degrading enzymes to migration and invasion, and quantitative real-time reverse transcriptase PCR (qRT-PCR) was used to assess changes in their expression.

**Results:**

Vinculin- and F-actin-rich lamellae were prominent in untreated and IL4-treated microglia (but not after LPS). IL4 increased the migratory capacity of microglia but eliminated the preferential anterior nuclear-centrosomal axis polarity and location of the microtubule organizing center (MTOC). Microglia degraded fibronectin, regardless of treatment, but LPS-treated cells were relatively immobile and IL4-treated cells invaded much more effectively through Matrigel™. For invasion, untreated microglia primarily used cysteine proteases, but IL4-treated cells used a wider range of enzymes (cysteine proteases, cathepsin S and K, heparanase, and matrix metalloproteases). Untreated microglia expressed MMP2, MMP12, heparanase, and four cathepsins (B, K, L1, and S). Each activation stimulus upregulated a different subset of enzymes. IL4 increased MMP2 and cathepsins S and K; whereas LPS increased MMP9, MMP12, MMP14 (MT1-MMP), heparanase, and cathepsin L1.

**Conclusions:**

Microglial cells migrate during CNS development and after CNS damage or disease. Thus, there are broad implications of the finding that classically and alternatively activated microglia differ in morphology, cytoskeleton, migratory and invasive capacity, and in the usage of ECM-degrading enzymes.

## Introduction

In the healthy adult brain, microglial cells continually extend and retract their ramified processes without overall cell displacement [1]. However, in the uninjured brain, microglia are highly migratory during the perinatal period of development. After central nervous system (CNS) injury in the adult, microglia retract their processes, adopt an amoeboid shape, and can migrate over relatively long distances to accumulate at damage sites [2,3]). In general, when cells migrate on a two-dimensional (2-D) substrate, they are polarized along the axis of movement, with a fan-shaped lamella bearing thin F-actin-rich protrusions (lamellipodia, filopodia) at the leading edge [4]. The forward-propelling machinery for cell migration requires turnover of substrate adhesions - with disassembly at the rear and re-assembly in newly protruded sites - while cell invasion through tissue also requires dissolution of the extracellular matrix (ECM).

When microglia respond to CNS damage or disease, it is expected that their activation mechanisms and outcomes will depend on the type of injury and stimuli encountered, for example, sterile versus non-sterile inflammation [5,6]. Part of the ongoing controversy about whether microglial activation is harmful or helpful in the damaged or diseased CNS [7,8] derives from their potential to exist in multiple activation states [6,9]. Until recently, models of microglial activation were based on macrophage activation, which was often simplified to classical (‘M1’) activation, evoked by exposure to interferon-γ or bacterial toxins (for example, lipopolysaccharide, LPS), and alternative activation (‘M2’), which is evoked by interleukin (IL)4 or IL13 [10-12]. Based on *in vitro* studies of microglia [13-17], it is clear that LPS can upregulate pro-inflammatory cytokines (for example, IL1β, tumor necrosis factor-α), excitatory amino acids, proteases, and reactive oxygen and nitrogen species. Exposure to LPS can inhibit neurogenesis [18] and exert neurotoxic effects *in vitro*[14,19,20] and *in vivo*[21]. Conversely, alternative activation, often characterized by increases in hallmark genes such as arginase 1 and the mannose receptor C type 1 (MRC1/CD206), is thought to help resolve acute inflammation by antagonizing pro-inflammatory mediators, initiating repair and reconstructing the ECM. Both IL4-stimulated macrophages and microglia generally produce less nitric oxide and more L-proline and type 2 cytokines (for example, IL10, transforming growth factor-β) that help promote tissue repair [5,6]. There is evidence that IL4-treated microglia promote neuroprotection [22,23], neurogenesis and oligodendrocyte genesis [24]. It is increasingly recognized that responses of microglia to CNS injury are more complex than M1 and M2 macrophage activation, and are likely modulated by the type of injury, timing and environment; possibly involving a continuum of states [9,25].

Here, as in numerous papers, to model the two extremes of microglial activation *in vitro*, we use LPS to induce classical activation and IL4 to induce alternative activation. The purpose of this study was to analyze how these activation states affect microglial migration, invasion, and the enzymes used for ECM degradation *in vitro*. We compared morphological hallmarks of migrating cells (F-actin distribution, cellular adhesions, orientation of the nuclear-centrosomal (NC) axis), and quantified random migration, chemotaxis in response to adenosine triphosphate (ATP), and invasion through Matrigel™. Finally, we compared microglial expression of nine matrix-degrading enzymes in three classes (heparanase, matrix metalloproteases (MMPs), and cathepsins), and used a panel of inhibitors to address their contributions to invasion. Because microglia migrate *in vivo* after many types of damage and disease, we initially expected that they would migrate and invade well, regardless of their activation state. Instead, our results show that microglial morphology, migration, invasion, and matrix-degrading enzyme usage differed depending on the activation state.

## Materials and methods

### Cell cultures

All procedures on animals were approved by the University Health Network Animal Care Committee, in accordance with guidelines from the Canadian Council on Animal Care. Our standard protocols [14,19,26] were used to isolate and culture primary microglia from 1 to 2 day-old Sprague–Dawley rat pups (Charles River, St.-Constant, PQ, Canada). Most importantly, these methods produce ≥99% pure microglia, and greatly reduce their levels of spontaneous activation [16,27]. In brief, after removing the meninges, the entire brain is minced, centrifuged (300 ×g, 10 min), re-suspended in Minimal Essential Medium (MEM; Invitrogen, Carlsbad, CA, USA) with 10% fetal bovine serum (FBS; Wisent, St-Bruno, PQ, Canada), and 0.05 mg/ml gentamycin (Invitrogen), and seeded in tissue culture flasks. After 48 hr culturing at 37°C and 5% CO_2_, the cells were washed and cultured with 2% FBS for 4 to 5 days. The flasks were then shaken (2 to 4 hr, 65 rpm, 37°C, 5% CO_2_) and microglia were harvested, washed and plated on substrates and at densities appropriate for each assay.

### Chemicals

Classical activation was evoked using 10 ng/ml LPS from *E. coli* K-235 (Sigma-Aldrich, Oakville, ON, Canada), as before [16,27]. Alternative activation was evoked with 20 ng/ml recombinant rat IL4 (R&D Systems Inc., Minneapolis, MN, USA), as before [27]. For the transmigration and invasion assays, microglia were treated 1 hr after either stimulus with one of the following inhibitors. The broad-spectrum MMP inhibitor, GM6001 (EMD Millipore, Toronto, ON, Canada) has K_i_ values from 0.2 to 27 nM depending on the MMP, and the heparanase inhibitor, OGT 2115 (R&D Systems) has an IC_50_ of 0.4 μM. The cysteine protease inhibitor, E-64 (Sigma), was used to inhibit cysteine cathepsins (Cats), (IC_50_ = 1.4, 4.1, 2.5 nM for Cat K, S and L, respectively). The selective Cat S inhibitor (Z-FL-COCHO; EMD Millipore) has a K_i_ value of 185 pM, and the selective Cat K inhibitor I (1,3-Bis(CBZ-Leu-NH)-2-propanone (EMD Millipore) has a K_i_ of 22 nM. All inhibitory constants were according to the suppliers. Stock solutions were made in DMSO (GM6001, OGT2115, Cat inhibitors), sterile double distilled water (LPS, E-64) or sterile phosphate buffered saline (PBS) with 0.3% bovine serum albumin (IL4). For all inhibitors, aliquots were stored at −20°C. ATP was prepared just before use.

### Quantitative real-time reverse transcriptase polymerase chain reaction

To monitor gene transcript levels, 500,000 cells were seeded into each 35 mm culture dish (Sarstedt Inc., Montreal, QC, Canada), and our standard protocol was used, as recently described [16,27]. Gene-specific primers (Table 1) were designed using ‘Primer3Output’ [28]. After 24 hr treatment with LPS or IL4, total RNA was extracted from primary microglia using the TRIzol method (Invitrogen), followed by RNeasy Mini Kit (QIAGEN, Mississauga, ON, Canada) for further purification. A two-step reaction was performed according to the manufacturer’s instructions (Invitrogen). In brief, total RNA (0.8 μg) was reverse transcribed in 20 μl volume using 200 U of SuperScriptII RNase reverse transcriptase, with 0.5 mM dNTPs and 0.5 μM oligo dT (Invitrogen). Amplification was performed on an ABI PRISM 7700 Sequence Detection System (PE Biosystems, Foster City, CA, USA) at 95°C for 10 min, 40 cycles at 95°C for 15 s, and 56°C for 20 s. ‘No-template’ and ‘no-amplification’ controls were included for each gene, and melt curves showed a single peak, confirming specific amplification. The threshold cycle (C_T_) for each gene was determined, and normalized to that of the housekeeping gene, hypoxanthine guanine phosphoribosyl transferase (HPRT1), which we find to be especially stable in primary rat microglia under all treatments we have investigated [16,19,27]. Results are expressed as relative mRNA expression (mean ± SEM) from four separate microglia cultures grown from four different rat pups.

**Table 1:** Primers used for qRT-PCR

**Gene**	**Genbank accession #**	**Primer sequences**
Cathepsin B	NM_022597.2	FP: CCCTGTGAACACCATGTCAATG
		RP: GATGTGGAGTAGCCAGCCTCAC
Cathepsin K	NM_031560.2	FP: TGTGGGTGTTCAAGTTTTTGCTG
		RP: GTACTGCTTCCCGTGGGTCTTC
Cathepsin L1	NM_013156.1	FP: ACCATGACCCCTTTACTCCTCCT
		RP: CTCCTCCACTCTTCCTCATTCGT
Cathepsin S	NM_017320.1	FP: CCATTCCTCCTTCTTCCTCTACCA
		RP: CCATCAAGAGTCCCATAGCCAAC
Heparanase	NM_022605.1	FP: GACGGACTGCTTTCCAAATCC
		RP: CGGGGAGAGGTTTTTCTGTTAGAG
HPRT1	XM_343829	FP: CAGTACAGCCCCAAAATGGT
		RP: CAAGGGCATATCCAACAACA
MMP2	NM_031054.2	FP: TCTCCCCCAAAACAGACAAAGAG
		RP: TCCTTCAGCACAAAGAGGTTGC
MMP9	NM_031055	FP: CTGCCTGCACCACTAAAGG
		RP: GAAGACGAAGGGGAAGACG
MMP12	NM_053963	FP: CTGGGCAACTGGACACCT
		RP: CTACATCCGCACGCTTCA
MMP14	NM_031056.1	FP: GTTCTGGCGGGTGAGGAATAAC
		RP: TCATAGGCAGTGTTGATGGATGC
TIMP-1	NM_053819	FP: GGTTCCCCAGAAATCATCG
		RP: GGAAACCTGTGGCATTTCC

*FP* forward primer, *RP* reverse primer.

### Immunocytochemical analysis

The methods were similar to our recent paper [29]. Microglia were seeded at 60,000 cells per UV-irradiated 15 mm glass coverslip (Fisher Scientific, Ottawa, ON, Canada). They were cultured for 1 day in MEM with 2% FBS, and then fixed in 4% paraformaldehyde (Electron Microscopy Sciences, Hatfield, PA, USA) at room temperature for 15 min. Cells were permeabilized with 0.2% Triton X-100 for 5 min and washed in PBS (3×, 5 min each). Non-specific binding was blocked with 4% donkey serum for 1 hr. All antibodies were diluted in 2.5% donkey serum and centrifuged before use (8200 ×g, 10 min) to precipitate aggregated antibody, if present. Microglia were incubated with a primary antibody overnight at 4°C: mouse monoclonal anti-vinculin (1:200; Sigma) or mouse monoclonal anti-α tubulin (1:1000; Abcam, Cambridge, MA, USA). Cells were washed (3×, 5 min each), blocked with 5% donkey serum for 1 hr, incubated with a corresponding donkey secondary antibody (1:200, Jackson Immunoresearch, West Grove, PA, USA) for 1 hr, and then washed (3×, 10 min each). Negative controls were prepared using the same protocol, but omitting primary antibody. Filamentous (F-) actin was visualized by incubating cells (15 min, room temperature) with Alexa Fluor 488-conjugated phalloidin (Invitrogen) at 1:50 in blocking solution. Cell nuclei were labeled with 4′,6-diamidino-2-phenylindole (DAPI; 1:3000 in PBS, 5 min; Invitrogen). After washing (3×, 5 min each), cells on coverslips were mounted on glass slides with Dako mounting medium (Dako, Glostrup, Denmark) and stored at 4°C. Microglia were sometimes labeled with FITC-conjugated tomato lectin (TL; 1:500, 15 min; Sigma), which binds to N-acetyl-lactosamine residues on the microglia surface.

Differential interference contrast (DIC) images were acquired with a Zeiss Axiovert 200 M microscope equipped with an ORCA-ER camera (Hamamatsu Corporation, Bridgewater, NJ, USA). All other images were acquired with either an LSM 510 META laser scanning confocal microscope or an Axioplan 2 widefield epifluorescence microscope equipped with an Axiocam HRm digital camera, and were analyzed with Axiovision 4.6 software (all from Carl Zeiss, Jena, Germany) or with ImageJ [30]. For many images, we acquired Z-stacks through the entire cell from high magnification epifluorescence images recorded at 200 nm increments. These images were then deconvolved using either Axiovision software with correction for Dako Fluorescent Mounting Medium or AutoQuant X software (version 2.2.2, Media Cybernetics Inc, Bethesda, MD, USA) using a theoretical point spread function and the constrained iterative algorithm. When constructing Z-stacks, the automated correction algorithm was used to compensate for fluorescence decay during repeated exposures. Cell auto-fluorescence and non-specific staining were monitored on cells exposed to secondary antibodies alone, with the same imaging and acquisition settings. This background was subtracted.

### Migration, substrate degradation and invasion assays

For the scratch wound assay, 80,000 cells were seeded onto each UV-irradiated 15 mm glass coverslip (Fisher Scientific) in 12-well plates. For transmigration and invasion assays, 30,000 cells were seeded onto each Transwell™ filter insert (VWR, Mississauga, ON, Canada). These methods are essentially the same as our recent papers [26,29], and will be stated only briefly here.

#### Scratch wound migration assay

One hour after plating the microglia, the standard medium (MEM with 2% FBS) was added. One hour later, LPS (10 ng/ml) or IL4 (20 ng/ml) was added. The cells were cultured for approximately 18 hr, at which time they were approximately 80% confluent. The monolayer was scratched with a sterile 200 μl pipette tip, and the cells were incubated for a further 24 hr to allow time for migration into the cell-free area. We counted all microglia in the scratch region and calculated the mean from five separate cultures.

#### Transmigration analysis

Microglia were suspended in standard medium, and 30,000 cells were added to the upper well of each Transwell™ insert (VWR), which bore an uncoated filter with 8 μm diameter holes. The lower well contained only medium. After 1 hr, microglia were incubated for 24 hr (37°C, 5% CO_2_) with either 10 ng/ml LPS or 20 ng/ml IL4. For the chemotaxis assay, 300 μM ATP (Sigma) was added to the lower well 1 hr after the addition of LPS or IL4. The cell-bearing filters were fixed in 4% paraformaldehyde for 10 min, rinsed with PBS, and the microglial cells remaining on the upper side of each filter were removed with a Q-tip. The filters were then stained with 0.3% crystal violet for 1 min, and again rinsed with PBS. The number of cells that had migrated to the underside was counted (5 random fields/filter) at 20× magnification using an Olympus CK2 inverted microscope (Olympus, Tokyo, Japan).

#### Fibronectin substrate degradation

A standard assay for degradation of ECM employs fluorescent-labeled substrate (usually fibronectin or gelatin) on glass coverslips. ECM degradation is then monitored as loss of the substrate fluorescence. We coated coverslips with HiLyte Fluor™ 488-labeled fibronectin (Cytoskeleton Inc., Denver, CO, USA) in PBS (2 μg/ml; 150 μl/coverslip). After 2 to 3 hr at 37°C, the fibronectin solution was aspirated off, microglia were added (50,000 cells/coverslip) and allowed to settle for 1 hr. Standard medium was added, followed 1 hr later by LPS (10 ng/ml) or IL4 (20 ng/ml). After a 24-hr incubation (37°C, 5% CO_2_), the cells were fixed and visualized using an Axioplan 2 widefield epifluorescence microscope equipped with an Axiocam HRm digital camera.

#### Invasion analysis

Microglial invasion was examined using BioCoat Matrigel™ Invasion Chambers (BD Biosciences, Mississauga, ON, Canada). These are similar to Transwell™ chambers, except that the 8 μm diameter holes in the upper filter are coated with Matrigel™, which is a basement membrane-type of ECM secreted by mouse sarcoma cells. Microglia in fresh standard medium (control) were added to the upper well (30,000/filter). After 1 hr incubation (37°C, 5% CO_2_), 10 ng/ml LPS or 20 ng/ml IL4 was added to the experimental wells. When used to stimulate chemotaxis, 300 μM ATP was added to the lower chamber of wells after another 1 hr incubation. All chambers were then incubated for 24 hr (37°C, 5% CO_2_).

### Statistical analysis

Quantitative data are presented as mean ± SEM, and analyzed with either one-way analysis of variance (ANOVA), followed by Tukey’s post-hoc test or two-way ANOVA with Bonferroni correction. GraphPad Prism ver 5.01 (GraphPad Software, San Diego, CA) was used. Results are considered significant if *P* <0.05.

## Results

### The microglial activation state affects their morphology

**Figure 1:**
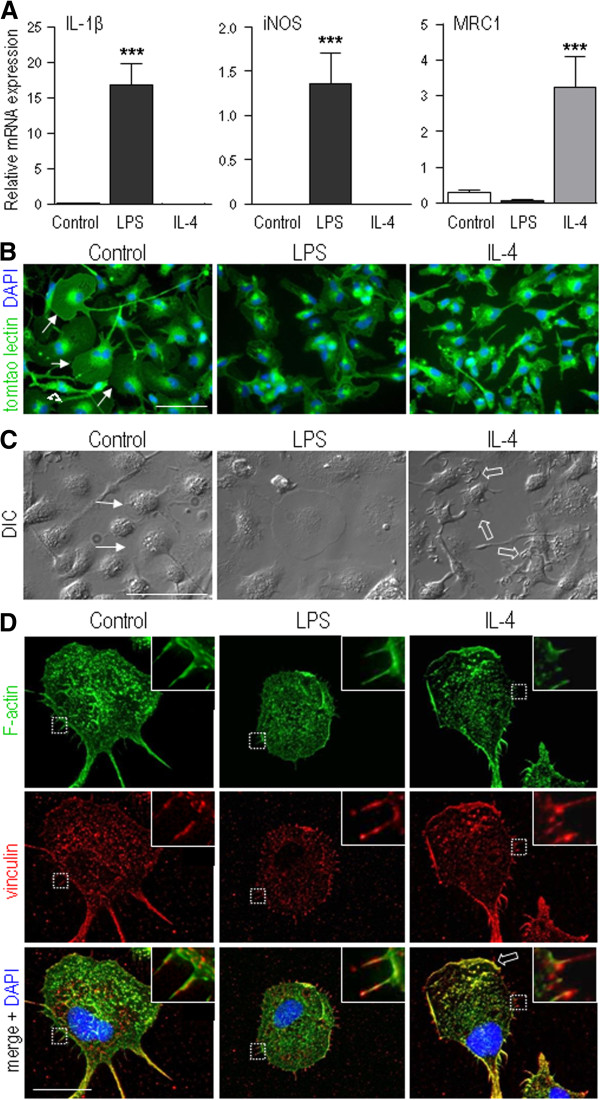
**The activation state affects microglial morphology. A**) Increased expression of molecules that are hallmarks of classical activation (iNOS, IL1β) and alternative activation (MRC1/CD206). Rat primary microglial cells were stimulated for 24 hr with 10 ng/ml lipopolysaccharide (LPS) or 20 ng/ml rat recombinant IL4. Gene expression was monitored by quantitative reverse transcriptase (RT)-PCR and normalized to the housekeeping gene, HPRT1. Values are expressed as mean ± SEM for four replicates using different cell cultures. One-way ANOVA with Tukey’s post-hoc test revealed differences from control microglia: ***P <0.001. **B**) Fluorescence micrographs show that the microglial morphology changes following stimulation with LPS or IL4. Fixed cells were stained with the microglia marker, tomato lectin (FITC-conjugated; green), and nuclei were visualized with DAPI (blue). Examples of unipolar microglia with a fan-shaped lamellum are indicated by arrows, and a bipolar cell is shown by the arrowhead. (Note the 100% purity of the microglial cultures.) Scale bar, 50 μm. **C**) Differential interference contrast (DIC) micrographs of microglia. For control, untreated microglia, the arrows indicate the smooth leading edge of the lamellum. For IL4-treated cells, the open arrows show regions of membrane ruffling. Scale bar, 50 μm. **D**) Higher-magnification deconvolved fluorescent images of microglia. The fluorescent labels show F-actin (stained with phalloidin, green), the actin-binding protein, vinculin (red), and cell nuclei (DAPI, blue). The boxed areas were digitally magnified (4×) and shown as insets. Scale bars, 20 μm (main images), 5 μm (insets).

In order to analyze functional outcomes of different activation stimuli, we have established culturing methods that maintain a relatively resting state with low production of cytokines and reactive oxygen and nitrogen species [14,16,27]. Here, untreated primary rat microglia had very low expression of the three activation markers: inducible nitric oxide synthase (iNOS), IL1β, mannose receptor 1 (MRC1/CD206). LPS selectively induced the classical activation markers, iNOS and IL1β, while IL4 selectively induced the alternative-activation marker, MRC1 (Figure 1A), as before [11]. We have used LPS extensively to investigate microglial responses that include gene expression [27], phagocytosis [16], and neurotoxic capacity [19], and have even compared different bacterial strains as sources of LPS [16]. Our experience is that 10 ng/ml LPS from *E. coli* strain K-235, as used here, is optimal for neonatal rat microglia, and induces numerous genes (for example, IL1β, TNFα, and iNOS) and functional responses (for example, NFκB and p38 MAPK activation, phagocytosis, and neurotoxicity) that are typical of a pro-inflammatory state. We chose the concentration of 20 ng/ml recombinant rat IL4 based on recent studies from our lab and others that found induction of well-known alternative activation markers [23,27] and neuroprotection [23].

The activation stimuli differentially affected the microglia morphology. Most untreated cells were unipolar, with a fan-shaped lamellum and one or more long processes (Figure 1B, C). A minority of cells was bipolar (Figure 1B, arrowhead). We previously showed that unipolar microglia are migrating in the direction of the lamellum and bipolar cells are not migrating, but microglia readily transition between migrating and non-migrating phenotypes [29]. Although the morphology was more variable after IL4 treatment, many cells were unipolar with a lamellum that was generally smaller than in control microglia (Figure 1B), and they exhibited extensive ruffling (Figure 1C, open arrows). After LPS treatment, most microglia were amoeboid shaped (Figure 1B) or round and flat (for example, Figure 1C, cell in center). Vinculin and F-actin staining were used to monitor the underlying cytoskeleton in deconvolved high-magnification fluorescent images (Figure 1D). Control cells had punctate vinculin and F-actin staining throughout the cell body and lamellum, with extensive co-localization in fine processes toward the trailing end (see insets). In IL4-treated cells, the vinculin and F-actin co-labeling was especially intense in the ruffles at the leading edge (open arrow) and in the uropod. LPS-treated microglia had short, fine vinculin- and F-actin-rich processes (inset) that lacked preferential orientation around the cell.

### Polarization of nuclear-centrosomal axis depends on the microglial activation state

**Figure 2:**
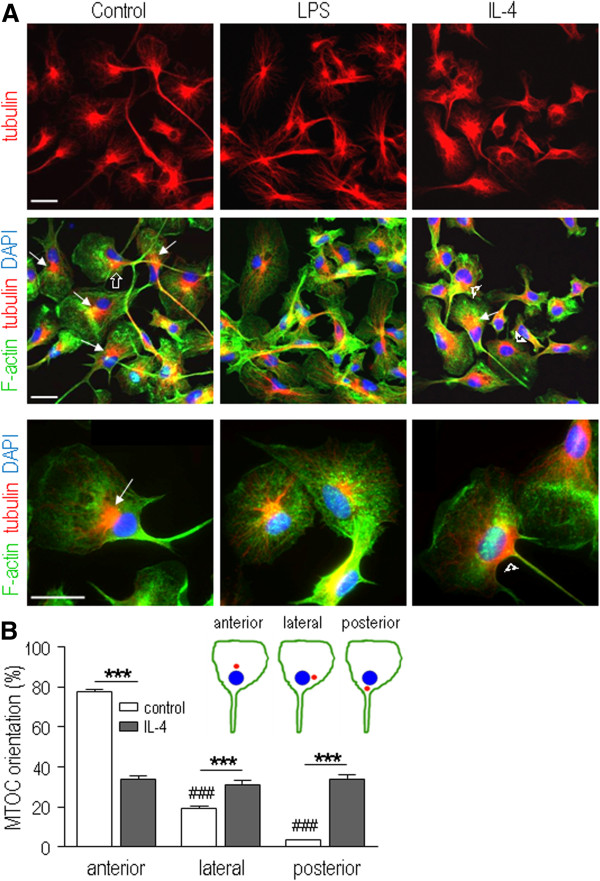
**Repolarization of the axis of the microtubule organizing center (MTOC) and nuclear centrosomal (NC) axis depends on the microglial activation state.** Rat microglia were stimulated for 24 hr with 10 ng/ml lipopolysaccharide (LPS) or 20 ng/ml rat recombinant IL4. **A**) Confocal images of microglia labeled for α-tubulin (red) to visualize microtubules and the MTOC, and DAPI (blue) to label nuclei. F-actin was labeled with Alexa Fluor 488-conjugated phalloidin (green) to reveal the cell shape. In unipolar microglia, the MTOC was in one of three peri-nuclear orientations (and see cartoon in panel **B**): toward the leading edge (arrows), at the side (open arrow) or toward the uropod (arrowheads). Higher magnification images (bottom panel) show the anterior and posterior positions, and the lack of microtubule polarization in LPS-treated cells. Scale bars, 20 μm. **B**) Quantification of MTOC-nuclear orientation. Only unipolar (migrating) microglia were analyzed: that is, control and IL4-treated cells. For each slide, ten random images were acquired at 10× magnification on the confocal microscope. MTOC orientation was scored based on its peri-nuclear position (cartoon inset): anterior (MTOC toward leading edge), posterior (MTOC toward uropod), lateral (MTOC at side). For each treatment replicate, ≥90 unipolar microglia were scored. Results are expressed as percent of total cells counted (mean ± SEM, n = 7 individual cultures). Two-way ANOVA with Bonferroni correction revealed significant intergroup differences: ***control differs from IL4-treated; ^###^MTOC position differs from anterior position of control cells. Three symbols indicate *P <*0.001.

When migrating on two-dimensional surfaces, many cell types (including macrophages), reorient the microtubule network toward the leading edge, so that the microtubule organizing center (MTOC) is anterior to the nucleus (see Discussion). As expected, in unipolar untreated microglia, the microtubules (labeled for α-tubulin) were dense near the nucleus, radiated toward the lamellum and fanned out, and were tightly bundled down the uropod (Figure 2A). A similar pattern was seen in unipolar IL4-treated cells. In contrast, the microtubule distribution in LPS-treated cells was less polarized, and they radiated toward the plasma membrane in multiple directions. We quantified the MTOC orientation in unipolar control and IL4-treated microglia with a prominent lamellum and a trailing uropod (Figure 2B). [LPS-treated cells were omitted because they did not display this morphology.] The cartoon illustrates the peri-nuclear MTOC positions: anterior, posterior, and lateral. Two scorers (one blinded to the treatments) independently quantified the data and obtained the same results. That is, under control conditions, the NC axis had reoriented in 77% of unipolar microglia to position the MTOC anterior to the nucleus, and only 3% of cells showed a posterior orientation. In striking contrast, in IL4-treated microglia, there was an equal likelihood of each of the three orientations.

### Migration, chemotaxis and invasion depend on the microglial activation state

**Figure 3:**
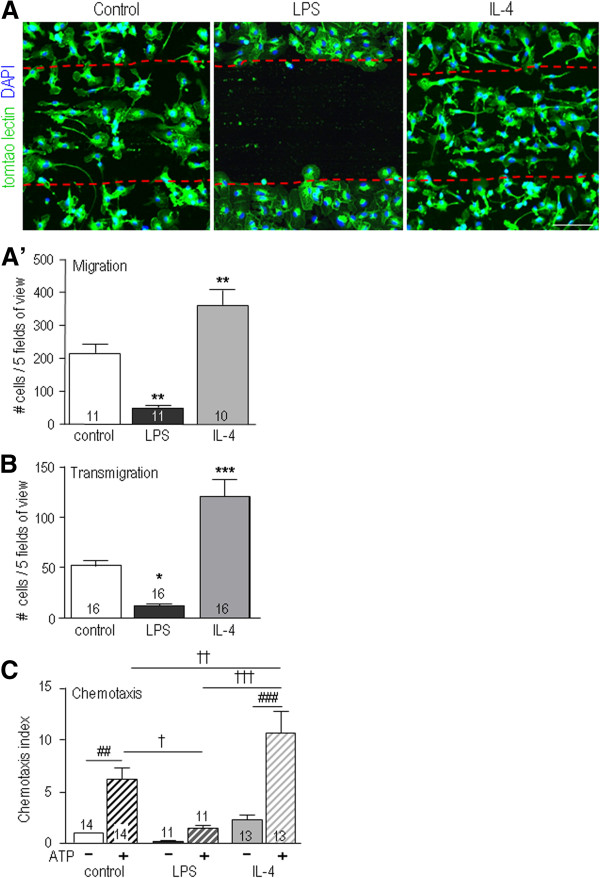
**Migration and chemotaxis are affected by the microglial activation state.** Cells were untreated (control) or exposed to 10 ng/ml lipopolysaccharide (LPS) or 20 ng/ml IL4. **A**) Migration into a scratch wound in a monolayer of microglia. Cells were fixed and stained after 24 hr with the microglial marker, FITC-conjugated tomato lectin (green), and the nuclear marker, DAPI (blue). Scale bar, 100 μm. **A**′) For each slide, confocal images of five random fields were taken along the border of the scratch (delineated by dashed red lines), and all lectin-positive cells within the scratch region were counted. **B**) Transmigration of microglia in Transwell™ chambers. After each 24-hr treatment, cells that had migrated to the underside of each filter were counted in five random fields. **C**) Chemotactic response to 300 μM ATP added to the lower Transwell™ chamber (striped bars) compared with unstimulated transmigration (solid bars). Results were analyzed as in panel B, and then normalized to control transmigration without ATP. Results are reported as mean ± SEM, with the number of individual cultures indicated on each bar. Statistical differences were determined using either one-way ANOVA with Tukey’s post-hoc test (**A, B**) or two-way ANOVA with Bonferroni post-hoc test (**C**). # indicates an effect of ATP; * (**A, B**) or † (**C**) indicates treatment differences (control *versus* LPS or IL4). One symbol, *P <*0.05; two symbols, *P <*0.01; three symbols, *P <*0.001.

Based on the observed differences in morphology and MTOC polarization (Figures 1 and 2), we hypothesized that the activation state will alter directional microglial migration. First, a scratch wound assay was used to analyze migration in 2-D while viewing the cell morphology (Figure 3A). Both untreated (control) and IL4-treated microglia migrated into the cell-free area but the response of IL4-treated cells was nearly 2-fold higher. Very few LPS-treated microglia migrated into the scratch wound (approximately 30% of the control value). Next, migration in 3-D was quantified using the Transwell™ chambers (Figure 3B). Significantly more IL4-treated microglia transmigrated than control cells (2.3-fold increase); whereas, LPS-treated cells migrated very little (24% of the control value). In all cases, transmigration was increased by a gradient of the chemoattractant, ATP (Figure 3C): that is, by 5.9-fold (control), 4.4-fold (IL-4-treated), and 7.3-fold (LPS-treated). Nevertheless, chemotaxis of IL4-treated cells remained the highest: 74% higher than control cells, 7-fold higher than LPS-treated cells.

**Figure 4:**
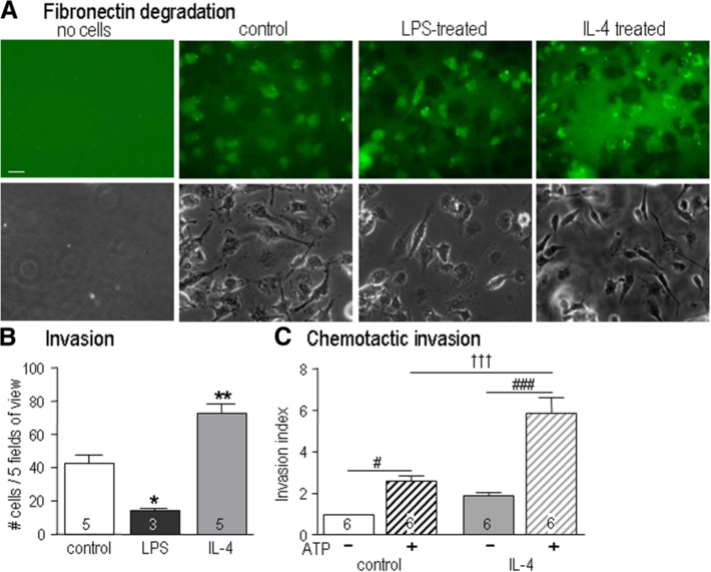
**The microglial activation state affects invasion through the extracellular matrix (ECM). A)** Microglia were plated on glass coverslips coated with fluorescent-labeled fibronectin, without (control) or after 10 ng/ml lipopolysaccharide (LPS) or 20 ng/ml IL4 treatment. The corresponding phase contrast images show cell locations and morphology. Scale bar, 20 μm. **B)** Microglia invasion through Matrigel™-coated 8 μm diameter holes in Transwell™ chambers. For each treatment, cells that had invaded to the underside of the filter after 24 hr were visualized and counted as in Figure 3B. Results are expressed as mean ± SEM for the number of individual cell cultures indicated on each bar. *P <0.05; **P <0.01. **C**) Chemotactic invasion. Either medium alone (solid bars) or the chemoattractant, 300 μM ATP (striped bars), was added to the lower well of the Transwell™ chamber. After 24 hr, cells were counted as in Figure 3C. Results were normalized to control invasion without ATP and reported as mean ± SEM for the number of individual cultures indicated. Statistical differences were determined using two-way ANOVA, followed by Bonferroni post-hoc tests. ### indicates effect of ATP; ††† indicates a difference between control and IL4-treatment. *P <*0.001.

We recently showed that unstimulated microglia can degrade fibronectin [26,29]. In the absence of microglia, the substrate fluorescence was uniform (Figure 4A). Regardless of treatment, microglial cells degraded fibronectin, leaving cell-sized patches of reduced fluorescence. The invasion capacity of microglia was then analyzed using an assay in which migration to the underside of each filter requires degradation of Matrigel™. IL4-treated microglia invaded 1.7-fold more than control cells; whereas, LPS-treated cells invaded 66% less (Figure 4B). Adding ATP to the lower well increased the invasiveness of unstimulated cells by 2.6 fold, and IL4-treated cells by 3.2 fold (Figure 3C). IL4-treated microglia had a 2.2 fold greater invasion capacity than unstimulated cells. LPS-treated cells were not analyzed because they migrated and invaded very poorly.

### IL4-treated microglia use a wide range of degradative enzymes for invasion

**Figure 5:**
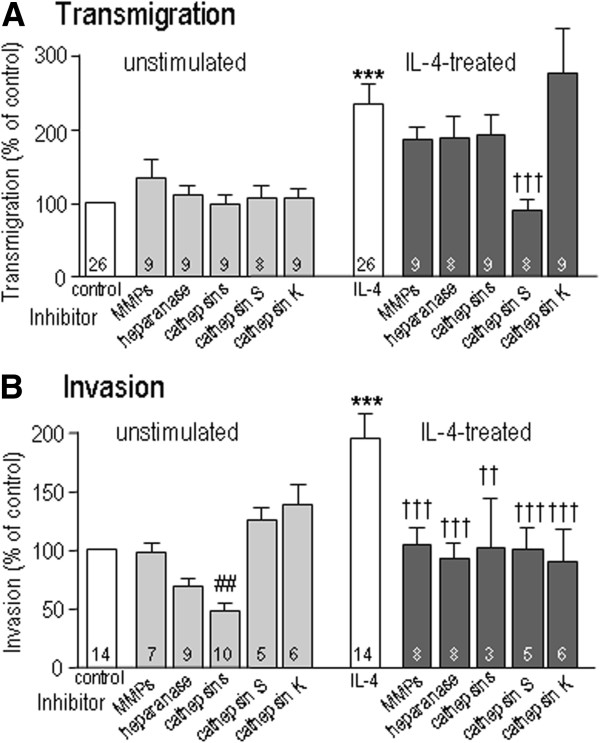
**Contributions of extracellular matrix (ECM)-degrading enzymes.** The enzyme inhibitors included three class-specific but broad-spectrum inhibitors: 5 μM GM6001 to inhibit MMPs, 5 μM OGT 2115 to inhibit heparanase, and the cysteine protease inhibitor, 10 μM E-64, which also inhibits cysteine cathepsins, including Cat B, K, L1 and S. The selective cathepsin inhibitors were: 5 nM Z-FL-COCHO to inhibit Cat S, and 250 nM 1,3-Bis(CBZ-Leu-NH)-2-propanone, also called Cat K inhibitor I. Microglia were untreated or exposed to 20 ng/ml IL4 for 24 hr. **A**) Transmigration through open 8-μm diameter holes in the filters of Transwell™ chambers. **B**) Invasion through Matrigel™-coated 8-μm diameter holes. *IL4-treated differ from control cells; #differs from untreated cells; †differs from IL4 alone. Two symbols, *P <*0.01; three symbols, *P <*0.001.

Degradation of ECM can involve any or all of three broad classes of degradative enzymes: MMPs, cathepsins, and heparanase. To analyze their contributions to microglia transmigration and invasion, we first used three class-specific but broad-spectrum inhibitors: GM6001 (MMPs), E-64 (cysteine proteases, including Cat B, K, L1 and S), OGT2115 (heparanase). Then, based on the results, we tested selective inhibitors of Cat S (Z-FL-COCHO) or Cat K (1,3-Bis(CBZ-Leu-NH)-2-propanone). For each inhibitor, we used a single concentration. Because the invasion assay was for 24 hr, during which the inhibitor efficacy might decrease, for the broad-spectrum inhibitors, we chose a high concentration in an attempt to inhibit all the subtypes within the relevant enzyme class. Then, for the selective Cat S and Cat K inhibitors we used a concentration that was 10 to 20 times the IC_50_, which is expected to inhibit >90% of the enzyme activity. Importantly, none of the inhibitors was toxic at concentrations and times used. For control, unstimulated microglia, none of the enzyme inhibitors affected transmigration through open holes in the filter (Figure 5A), which also demonstrates lack of non-specific effects or toxicity.

Only IL4-treated microglia were compared with controls because LPS-treated cells migrated very poorly (Figures 3 and 4B). IL4 treatment greatly increased transmigration, and this was reduced back to the control level by the Cat S inhibitor. The apparent trend toward reduced transmigration by three other inhibitors did not reach statistical significance with the sample size used. None of the inhibitors affected cell viability at the concentrations and times tested. Interestingly, invasion through the same ECM substrate (Matrigel™) required different enzymes in untreated and IL4-treated microglia. In unstimulated microglia, invasion was inhibited only by the broad-spectrum cysteine cathepsin inhibitor, E-64, which decreased invasion to approximately 50% below the control level (Figure 5B). Invasion was not altered by the selective Cat S and K inhibitors, suggesting that E-64 acts through a different enzyme. IL4 treatment increased invasion about 2-fold, and all the enzyme inhibitors then reduced it to the baseline level. These results demonstrate that IL4-treated microglia can use all three classes of ECM-degrading enzymes for invasion. Untreated microglia were more restricted, using primarily cysteine cathepsins.

### The microglial activation state alters expression of ECM-degrading enzymes

**Figure 6:**
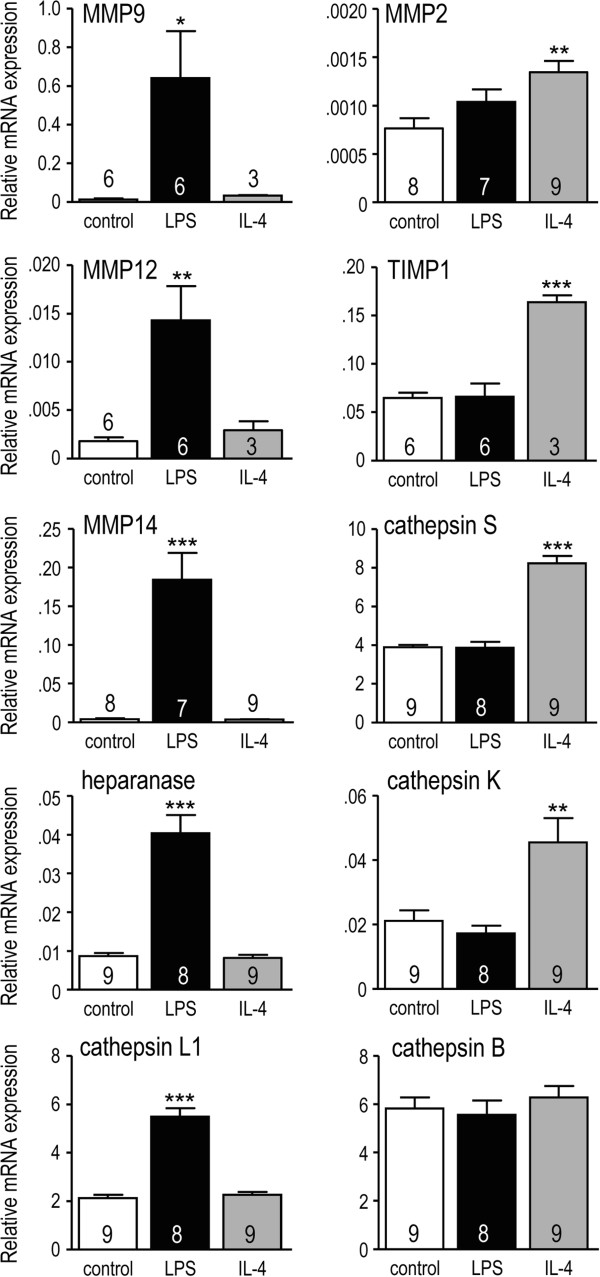
**Expression of extracellular matrix (ECM)-degrading enzymes.** Gene expression was monitored by quantitative reverse transcriptase (RT)-PCR and normalized to the housekeeping gene, *Hprt1*. (Note the different Y-axis scales.) The values are expressed as mean ± SEM, with the number of individual cultures indicated on each bar. The genes are: *Mmp2*, *Mmp9*, *Mmp12*, *Mmp14*, *Timp1*, *heparanase*, *Cat B*, *Cat L1*, *Cat S*, *Cat K*. One-way ANOVA with Tukey’s post-hoc test revealed differences from control microglia: *P <0.05, **P <0.01, ***P <0.001.

Based on the differences in migration and enzymes used for invasion in unstimulated versus IL4-treated microglia, we next compared transcript expression of several ECM-degrading enzymes (Figure 6). LPS-treated cells were also examined because they degraded fibronectin (Figure 4) despite being poorly migratory (Figure 3). For eight of the nine enzymes examined, the pattern was unique to the stimulus (there was no change in Cat B). LPS-treated microglia had increased MMP9, MMP12, MMP14 (MT1-MMP), heparanase and Cat L1. In IL4-treated microglia only MMP2, Cat S and Cat K increased (Figure 6), which is consistent with the unique contribution of Cat S and Cat K to invasion in IL4-treated cells (Figure 5). Given the small increase in MMP2 only, and the increase in the general MMP-inhibitor, TIMP metallopeptidase inhibitor 1 (TIMP1), we were surprised that invasion by IL4-treated cells was reduced by the broad-spectrum MMP inhibitor, GM6001.

## Discussion

We report the novel finding that IL4-treated, alternatively activated rat microglia have an increased migratory capacity in both 2-D (scratch wound) and 3-D (Transwell™) assays. We found that LPS-treated microglia were less migratory. Previous reports are inconsistent, and while the reasons are not clear, the effect of LPS on migration might depend on species and strain, cell type and age. Impaired migration has been reported for neonatal rat [31] and adult human microglia [32], and for guinea pig peritoneal macrophages [33] and rabbit alveolar macrophages [34]. Conversely, some studies reported that LPS can increase migration in the RAW264.7 macrophage cell line [35,36] and primary rat peritoneal macrophages, but the LPS dose was not stated [35]. Interestingly, migration of peritoneal macrophages was mildly inhibited by LPS in LPS-sensitive mouse strains (C3H/HeN, C57BL10/S) but increased in LPS-resistant mice (C3H/HeJ, C57BLlO/ScCR strains), although only at LPS doses greater than 50 ng/ml [37]. The effect of microglia age is also unknown; the rat microglia studies used neonates (present study; [31]), while the macrophage studies used adult animals. Another difficulty in comparing results is the differing concentrations and strains of LPS used but an earlier dosing study found that the same concentration (10 ng/ml) reduced migration of primary guinea pig peritoneal macrophages by 56% [33].

After acute CNS injury, damaged cells can release ATP, glutamate and nitric oxide, which can attract microglial processes [1] and recruit them to damage sites [2]. In all of our assays, ATP increased microglia migration, whether the cells were untreated or stimulated with IL4 or LPS. While this shows that microglia can respond to ATP regardless of their activation state, IL4-treated microglia remained the most migratory and LPS-treated cells the least migratory. In the damaged brain, additional stimuli and chemotactic factors (for example, chemokines and matricellular molecules) will be present and that their effects on migration patterns of activated microglia might be complex.

The migratory phenotype is established by interactions between a cell and substrate [38] and is often analyzed as 2-D migration on glass. The lamellum adheres to the ECM, provides a broad surface for traction, and contains a network of actin filaments, like that seen in untreated rat microglia. We found that the morphology and cytoskeletal arrangement of microglia was profoundly affected by LPS, and more subtly affected by IL4. LPS-treated cells were ameboid or rounded up, and had many vinculin-rich and F-actin-rich filopodia without a specific orientation. This is consistent with previous descriptions of LPS-activated microglia [39,40]. In contrast, most resting and IL4-treated microglia had a polarized morphology, with a lamellum at the front and a uropod at the rear. In earlier work, IL4 changed rat [41] and mouse [42] primary microglia from rounded or ameboid to a more ramified shape, with processes and lamellipodia. However, we found that the lamellum of IL4-treated cells was smaller and exhibited more membrane ruffles, and both the lamellum and uropod showed extensive co-localization of F-actin and vinculin. Changes in actin distribution and polymerization underlie the morphological polarization and roles of both the lamellum and the uropod [4]. Precise roles of the uropod in cell migration are unknown but it is considered important for cells that migrate through tight spaces [38,43]. The presence of a uropod and lamellum in resting and alternatively activated microglia suggests that these cells will migrate well through the tightly packed brain parenchyma during development and after CNS injury.

A hallmark of polarization in migrating cells is coordinated reorientation of the NC axis [44]. In many migrating cells, the nucleus moves toward the rear, resulting in an ‘anterior’ NC axis in which microtubules oriented toward the leading edge are stabilized. The MTOC, endoplasmic reticulum and Golgi apparatus are then in front of the nucleus. Many cells display an anterior NC orientation when migrating on 2-D substrates: for example, macrophages, neurons, astrocytes, and epithelial and mesenchymal cells [44]. The opposite ‘posterior’ NC orientation (nucleus in front of MTOC) is less common but seen in some migrating immune cells, especially neutrophils [45] and T lymphocytes [44]. The precise role of the MTOC position in cell migration is unknown; however, it can be affected by extracellular cues. For instance, neutrophils changed their MTOC orientation to an anterior position during chemotaxis [46], and to a dorsal position near the cell surface after exposure to an antigen-antibody complex [47]. MTOC repositioning during non-migratory events includes re-orientation toward phagosomes in macrophages [48] and toward the immune synapse in bone-derived dendritic cells [49]. Neutrophils are especially interesting because they are one of the fastest moving mammalian cells [50], and exhibit a variable MTOC orientation during random migration on glass [45] or formvar (a support film for electron microscopy) [46]. We found that the MTOC in untreated microglia was polarized toward the leading edge; whereas, the highly migratory IL4-treated cells lacked this preferential MTOC/NC orientation. IL4-treated microglia also had a smaller lamellum than control cells, with extensive membrane ruffling that is consistent with reduced adhesion. LPS-treated microglia were much less migratory, lacked a lamellum and uropod and had many filopodia, suggesting that they adhere more tightly to the substrate.

Cell invasion requires migration and substrate degradation. Specifically, in order to navigate the tightly packed brain parenchyma *in vivo*, microglia need to cleave cell-substrate interactions and degrade the ECM. Given the dramatic changes in microglial migration evoked under different activation conditions, it was important to determine if cell invasion was affected, and if so, whether the expression and roles of specific matrix-degrading enzymes were altered. We observed that rat microglia could degrade fibronectin regardless of their activation state but their ability to invade through Matrigel™ differed dramatically. IL4-treated microglia invaded more than untreated cells, and LPS-treated microglia invaded less. While differences in their migratory capacity contribute, this cannot account for the different matrix-degrading enzymes used for invasion by untreated versus IL4-treated microglia. Migration of untreated microglia on 2-D substrates did not require any of the enzymes tested. In contrast, IL4-treated cells used a broad range of enzymes for migration and especially for invasion through ECM. Importantly, in untreated microglia, we found that the heparanase inhibitor reduced invasion through Matrigel™, which supports a role for heparanase in ECM degradation. This is consistent with a study reporting that heparanase is involved in invasion of untreated microglia [51]. In that study, LPS evoked an increase in the active heparanase isoform and degradation of heparan sulfate proteoglycans.

Expression of almost all matrix-degrading enzymes examined differed with the microglial activation state. There are previous reports that microglia express heparanase, as well as several MMPs and cathepsins [52]. Little is known about how LPS alters their expression, and almost nothing is known about the effect of IL4. We found that IL4 treatment uniquely upregulated several constitutively expressed enzymes: MMP2, Cat K, Cat S, and the MMP inhibitor, TIMP1. LPS uniquely up-regulated MMP9, MMP12, MMP14 (MT1-MMP), heparanase and Cat L1, but did not alter MMP2, TIMP1 or Cat -B, -K or -S. Previously, LPS was seen to increase expression of MMP12 and MMP14 in human microglia [52], and MMP9 and MMP14 in murine microglia [53]. Given the broad range of enzymes expressed by LPS-treated cells, their poor invasion capacity was likely due to the lack of migration capacity.

It is an intriguing finding that microglia expressed and used different cathepsins for migration and invasion: especially Cat S in IL4-treated cells. Most cysteine cathepsins are lysosomal endopeptidases that are active only at acidic pH but Cat S is enzymatically active at both acidic and neutral pH [54] and can degrade some ECM components of the CNS [55]. Some cathepsins are ubiquitously expressed (Cat B, Cat L) and others are more cell-specific (Cat K in osteoclasts) [56]. Cat S is thought to be restricted to antigen-presenting cells [57] and can be secreted by macrophages and microglia [54,58]. Cat S is expressed in unstimulated microglia (present study) and is induced in microglia following spinal cord injury, where it contributes to neuropathic pain [57]. There are several previous studies of microglia activation and Cat S but the results are inconsistent, and information relating it to IL4 treatment is very limited. IL4 increased the Cat S activity in tumor-associated macrophages [59], and we found it selectively upregulated Cat S (and Cat K) expression. Cat S was involved in microglial migration and invasion; whereas, Cat K was only needed for substrate degradation and invasion, consistent with its essential role in bone resorption by osteoclasts [60]. After LPS treatment of primary rat microglia, we saw no change in Cat S expression. Several studies have used microglia cell lines, and this might account for the discrepancies seen. Using the murine N-13 microglial cell line, one study reported that LPS decreased Cat S cellular levels and activity but increased its secretion [58], and another showed that basic fibroblast growth factor increased both intra- and extracellular Cat S activity [54]. In the BV-2 microglia cell line, LPS increased intracellular levels of Cat S and Cat X but evoked secretion of Cat -B, -K, -S and -X [61]. Interestingly, co-stimulation of the P2X7 purinergic receptor was necessary for secretion of enzymatically active Cat S from LPS-treated rat primary microglia [62]. While there is limited information about the roles of Cat S *in vivo*; based on its actions on T cell polarization, Cat S inhibitors are being considered for use in autoimmune diseases [63].

## Conclusions

Microglia migrate during normal CNS development and after disease or damage in the adult. Their functional roles will depend on their activation state, which itself is modulated by complex environmental cues. Classical and alternative activation states have been identified for microglia (and macrophages) and are associated with generally damaging and reparative functions, respectively. Regardless of their activation state, microglia must migrate and degrade the dense ECM to reach their target site. Thus, it is significant that classically and alternatively activated microglial cells differed in their capacity for migration and invasion, and in levels and usage of several matrix-degrading enzymes *in vitro*. These differences might determine how well they reach target sites, and by providing specificity in matrix degradation, potentially reduce bystander damage to the healthy ECM.

## Abbreviations

CNS: Central nervous system; DIC: Differential interference contrast; ECM: Extracellular matrix; LPS: Lipopolysaccharide; MTOC: Microtubule organizing center; NC: Nuclear-centrosomal.

## Competing interests

The authors declare that they have no competing interests.

## Authors’ contributions

SL designed and carried out the functional studies and immunohistochemistry, and analyzed the data. LCS obtained funding, helped conceive the project and interpret results. Both authors prepared, read, and approved the final manuscript.
